# *QuickStats:* Percentage* of Adults Aged 20–64 Years With a Fasting Test in the Past 12 Months for High Blood Sugar or Diabetes,**^†^** by Race/Ethnicity**^§^** — National Health Interview Survey,**^¶^** United States, 2011 and 2016

**DOI:** 10.15585/mmwr.mm6647a7

**Published:** 2017-12-01

**Figure:**
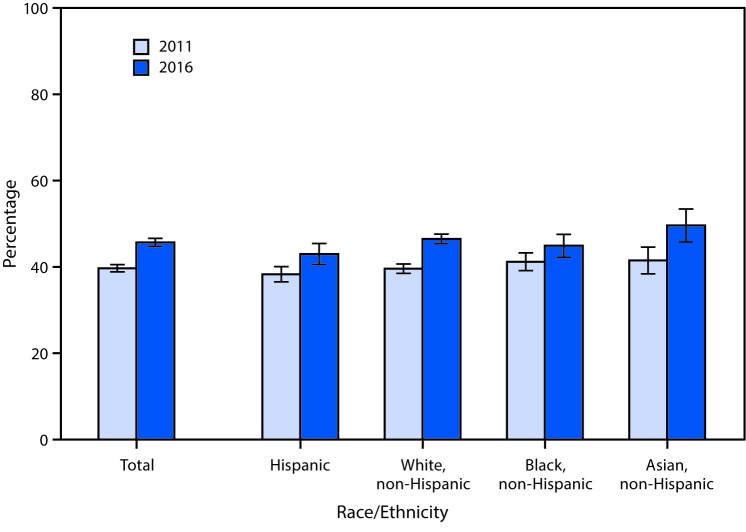
The percentage of U.S. adults aged 20–64 years who had a fasting test for high blood sugar or diabetes in the past 12 months increased from 39.7% in 2011 to 45.7% in 2016. From 2011 to 2016, there was an increase in the percentage for all racial/ethnic groups examined: Hispanic (38.3% to 43.0%), non-Hispanic white (39.6% to 46.5%), non-Hispanic black (41.2% to 44.9%), and non-Hispanic Asian (41.5% to 49.6%) adults. In 2011, there was no statistically significant difference among the four groups examined, but in 2016, Hispanic adults were less likely than non-Hispanic white and non-Hispanic Asian adults to have had a fasting test, and non-Hispanic Asian adults were more likely than non-Hispanic black adults to have had one.

**Source:** National Health Interview Survey, 2011 and 2016 data. https://www.cdc.gov/nchs/nhis.htm.

